# Editorial: Resolving the complexity of plant genomes and transcriptomes with long reads, volume II

**DOI:** 10.3389/fpls.2023.1326500

**Published:** 2023-11-16

**Authors:** Agnieszka Zmienko, Paweł Wojciechowski, Marek Figlerowicz

**Affiliations:** ^1^ Institute of Bioorganic Chemistry of Polish Academy of Sciences, Poznan, Poland; ^2^ Faculty of Computing and Telecommunications, Institute of Computing Science, Poznan University of Technology, Poznan, Poland

**Keywords:** long read DNA sequencing, assembly, transcriptomics, transposable element (TE), PacBio, nanopore

It has been over 20 years since the genome assembly of the first land plant – *Arabidopsis thaliana* – has been published (Arabidopsis Genome Initiative, 2000). During this time, numerous plant genomes have been sequenced and large-scale studies of intra- and interspecific genetic variation were conducted. Many plant genomes are large and complex, with varying levels of ploidy and/or high abundance of the transposable elements (TEs), which has been a strongly limiting factor in genomic studies. However, within just a decade since the emergence of long read sequencing (a.k.a third generation sequencing) we have observed a dynamic increase in the number of *de novo* assembled genomes in the public databases. This could have not been achieved without the impressive improvement of the PacBio HiFi and Nanopore sequencing, which are currently the most popular third generation sequencing methods. Both techniques deliver reads with >99% base accuracy and the accuracy of PacBio HiFi reads, which are generated by circular consensus sequencing, is comparable to that of the classical Sanger sequencing. On the other hand, Nanopore sequencing offers unrestricted read length, with >4 million base already reported. Accordingly, the range of analyzed species quickly expanded beyond the most common or economically important ones, promising unprecedented insight into the genetic biodiversity on Earth (Gupta, 2022). In line with this trend, within this Research Topic we present studies which utilized long read sequencing to investigate pant genomes, with the special emphasis on producing high quality *de novo* assemblies of species unique to specific geographical localizations or habitats. This included two trees from Fagaceae family, Chinese cork oak (*Quercus variabilis*) and Japanese chestnut (*Castanea crenata* Sieb. et Zucc), which are ecologically and economically important species native to East Asia (Han et al, Wang et al.), as well as four tree species representing *Syzygium* genus (Myrtaceae), three of them being autotetraploid (Ouadi et al.). The assemblies were compared with the available genomes of related plants, which provided valuable insight into the genome evolution and the history of gene family expansions/contractions in the species of interest.

Another interesting study reported assembling the genome of Tibetan sea-buckhorn (*Hippophae tibetana*). This perennial dense thorny shrub can be found in Tibetan Plateau, which is the world’s highest and largest plateau. The extreme environmental conditions in this region impose strong selective pressures and drive genome evolution, leading to unique adaptations of the local species. Wang et al. sequenced the Tibetan sea-buckhorn genome to study its organization and search for the genetic features that might contribute to its ability of growing at high altitudes – even 5000 meters above see level. They found that TE amplification largely accounted for its genome-size expansion. Moreover, based on the comparison of the relative position of genes and intact or fragmentary Gypsy/Copia elements, they suggested that these retrotransposons might specifically play a role in adaptation to high-altitude habitats. Interestingly, Ouadi et al. also observed species-specific evolutionary dynamics of Gypsy and Copia retrotransposons in the *Syzygium* species. Repetitive elements also constituted a substantial fraction of the Chinese cork oak and Japanese chestnut assemblies (67.6% and 58.78%, respectively), which highlights the important role of TE amplification in shaping plant genomes.

Along with the lowering costs and increased access to the sequencing devices, many labs incorporated genome sequencing as the first step in functional genomic studies. A nice example of such approach has been presented by Hu et al., who produced the genome of *Scutellaria baicalensis* Georgi (Lamiaceae) with third generation sequencing reads and used it to annotate genes. Next, they quantified and compared gene expression among the plants differing by the color of flowers (purple, pink or white). The roots of *S. baicalensis* are rich in flavonoids, therefore the authors focused especially on characterizing and comparing the expression of genes involved in flavonoid biosynthesis pathway and transcription factors related to this process.

The genomes of plants described in this Research Topic’s papers have different sizes, from about 380 Mb in *Syzygium* and *S. baicalensis* up to 1.5 Gb in Tibetan sea-buckhorn. It should be stressed that in all presented cases, the draft genomes (either based on the PacBio or Nanopore reads), were scaffolded with the additional information from the chromosome conformation capture (Hi-C) data. Indeed, combining third generation sequencing with the methods allowing to determine chromatin structure or with high density genetic maps has proven to be invaluable in assembling telomere-to-telomere chromosomes of large plant genomes, e.g. watermelon and maize (Deng et al., 2022; Chen et al., 2023). With the fast improvement of the Nanopore reads length and accuracy as well as the development of bioinformatics algorithms, we can expect complex gap-free genomic assemblies resolved solely with long read sequencing data in the near future.

The possibility to obtain full-length cDNA sequences with third generation sequencing creates fantastic opportunities also for transcriptomic studies. Long read-based *de novo* transcriptome assembly and quantification facilitates identification of gene UTRs and distinction between the alternatively spliced transcripts, representing different protein isoforms. It is also much easier to unambiguously map long reads to genes from which they were derived. It seems especially important for plant genomes, which frequently undergo whole genome and segmental duplication events. Hu et al. used long read approach to investigate the transcriptomic complexity of 9 citrus species and their close relatives. Using the Nanopore data they were able to identify both novel isoforms of known genes as well as new expressed genes in all analyzed species. Having full-length transcript sequences also allowed them to analyze the frequency of various types of splicing events. Additionally, they identified long noncoding RNAs in their datasets.

Long read sequencing has been named the Method of the Year 2022 (Marx, 2023). With the growing number of providers and applications, including DNA methylation analyses and single-cell long read sequencing, it seems certain that this technique will irrevocably and positively influence the genomic studies of the entire decade.

## Author contributions

AZ: Conceptualization, Writing – original draft, Writing – review & editing. PW: Conceptualization, Writing – review & editing. MF: Conceptualization, Writing – review & editing.
